# The Experiences of Family Members of Ventilated COVID-19 Patients in the Intensive Care Unit: A Qualitative Study

**DOI:** 10.1177/10499091211006914

**Published:** 2021-04-01

**Authors:** Chiahui Chen, Elaine Wittenberg, Suzanne S. Sullivan, Rebecca A. Lorenz, Yu-Ping Chang

**Affiliations:** 1 School of Nursing, 12292 University at Buffalo–The State University of New York, Buffalo, NY, USA; 2 Department of Communication Studies, 14669 California State University Los Angeles, CA, USA

**Keywords:** COVID-19, intensive care units, family, communication, palliative care, end-of-life

## Abstract

**Background:**

Visitor restrictions caused challenges for family members when their loved ones had coronavirus disease (COVID-19) and were ventilated. Limited studies have reported on family members’ experiences and support needs.

**Aim:**

To explore the experiences and support needs of family members of ventilated COVID-19 patients in the intensive care unit (ICU).

**Design:**

Exploratory, qualitative design, using in-depth individual telephone interviews, and analyzed using thematic analysis.

**Setting/Participants:**

Ten family members of adult COVID-19 patients in the ICU.

**Result:**

Seven key themes represented family members’ experiences: (a) reactions to the COVID-19 diagnosis, (b) COVID-19 as a destabilizing force on the family unit, (c) COVID-19’s effects on bereavement outcomes, (d) desperately seeking information, (e) family member needs, (f) conflicting feelings about video calls, and (g) appreciation of care. Family members’ feelings about the patient’s diagnosis and how the virus was contracted exacerbated their stress and anxiety. They struggled to feel informed about care that they could not witness and had difficulty understanding information. Family members reported that video calls were unhelpful. While these experiences made them question the quality of care, they expressed their appreciation of the frontline healthcare providers taking care of their loved ones.

**Conclusion:**

The stress and uncertainty of family members of critically ill patients with COVID-19 were influenced by their inability to feel connected to the patient and informed about care. Healthcare providers should assess each individual family’s burden and preferences, and this should include establishing structured, timely, and consistent communication regarding patient care during the pandemic including early referral to palliative care.

## Introduction

Family members play an essential role in the intensive care unit (ICU) because critically ill patients rely on them to make surrogate decisions and communicate their needs. Known challenges for family members in the ICU include (a) low health literacy leading to a lack of understanding of health care information required to make informed health decisions,^1^ (b) dissatisfaction with communication,^2^ (c) delayed negative prognostic communication at end-of-life (EOL),^3^ (d) immense emotional distress, such as post-traumatic stress disorder (PTSD), and (e) complicated grief.^4^ Since the first coronavirus disease 2019 (COVID-19) case was reported in January 2020 in Washington State, there have been 26,087 ICU admissions for COVID-19 with more than 29 million cases and 524,695 deaths in the United States.^5,6^ In order to minimize the risk of potential COVID-19 transmission, visitor restriction policies were quickly and aggressively instituted in many hospitals.^7^ The COVID-19 pandemic has not only caused imminent threat to the infected patients’ life, but it also created additional barriers for communication between healthcare providers and family members who are no longer able to be at the bedside. We conducted a qualitative study using semi-structured individual interviews to further our understanding about the effect of the visitation restrictions on families of ventilated COVID-19 ICU patients.

## Method

### Design

An exploratory qualitative design was guided by the Universal Model of Family-Centered Care.^8^ Data collection included 2 audio-recorded, in-depth individual telephone interviews. The first interviews were scheduled with the recruited family members at their preferred time while their loved ones were in the ICU, and the second interviews were scheduled 4-6 weeks after the ICU discharge to explore any sustained impact of the experience.

### Setting/Sampling

Participants were recruited from a medical ICU in an academic tertiary hospital, located in Buffalo, New York, United States. The individual telephone interviews were conducted in a private room to maximize privacy and reduce interruptions. Purposively sampled family members meeting inclusion criteria (Table 1) were invited to participate in the study. Participant recruitment ended when data saturation was attained.

**Table 1.** Inclusion/Exclusion Criteria.

Inclusion criteria	Exclusion criteria
Patients	Patients
Age ≥ 18 years COVID-19 diagnosis Mechanically ventilated In the ICU for ≥ 24 hours	Deceased or discharge from ICU within 24 hours Able to communicate and make own decision Lack of family members or any other significant person
Family members	Family members
Age ≥ 18 years Be a first degree relative or any other person who self-identifies as significant to the patient (e.g., parents, spouse, significant others, children, and siblings) Having been listed in the medical record as the person of contact for the patient	Not listed in the medical record as the person of contact for the patient Not able to complete consent process and questionnaires in English

### Data Collection/Procession

The prospective eligible participants (family members) were approached by telephone (C.C.) regarding their interest in study participation. If interested, details of the study were discussed and questions were answered. Written or verbal consent was obtained before the interview. Given the seriousness and sensitivity of the topic, all participants received information on mental health resources at the time of enrollment. Prior to conducting individual interviews, family members were asked to complete the demographic questionnaire (C.C.). Semi-structured telephone interviews of 30- to 45-minutes were held from May 2020 to August 2020 by the researcher (C.C.) who has 12 years of critical care nursing experience. All telephone interviews were audio-recorded, transcribed verbatim by Nuance® Dragon® Professional transcription software,^9^ and then checked for accuracy by the interviewer (C.C.). As data was being collected, the researchers (C.C. and S.S.) read and re-read the transcripts to get a general sense of the experience of being a family member of the COVID-19 ventilated patient in the ICU. The researchers (C.C. and S.S.) individually coded the transcripts by dividing the significant text segments into “meaningful units” keeping the participants’ own words. Next, they (C.C. and S.S.) discussed and compared all codes until no new concepts were emerging.

### Data Analysis

Following data saturation, 2 independent analysts (C.C. and E.W.) started line-by-line coding and writing memos on the transcripts. The analysis was driven by an inductive, concurrent, thematic analysis with constant comparisons. The process included (a) examining consensus codes, (b) identifying patterns, (c) creating categories to describe “critical moments,” (d) categorizing the codes into overarching elements and higher-level categories, and (e) clustering the similar categories into related themes. In order to make sure that the categories were useful and accurate representations of the data, the analysts (C.C. and E.W.) returned to further analyze the selected quotes and compared the categories against them. The last step of the analysis was to refine and name the themes by formulating their meanings and determining how the themes helped to create a further understanding of family members’ experiences when their loved ones were ventilated due to COVID-19.

### Rigor

In line with Gibbs’s (2018) recommendations, 3 steps were taken to increase reliability and validity of the findings.^10^ First, the data collection and analysis were consistent and reviewed across different researchers (C.C., S.S. and E.W.). Second, the interviewer (C.C.) who had experience providing care to the COVID-19 patients and their family members in the ICU guided the inquiry process to collect in-depth data. Third, the analysts (C.C. and E.W.) used participants’ own language at all levels of coding. The themes were developed based on converging feedback from participants, which added to the credibility of findings.

## Results

Thirty-one family members meeting eligibility criteria were invited to participate: 21 declined, 10 were interviewed once, and 3 were interviewed twice. The included 10 family members were of a median age of 51 years (range: 36-77 years), and 8 of whom were female (see Table 2). We had variation in ethnicity (4 Caucasian, 5 African American, and 1 Asian American) and relationship of the family member to the patient (3 spouses, 3 children, 2 parents, 1 nephew, and 1 niece). The number of patients who died during the ICU stay or immediately after ICU stay was 4 (40%). Seven key themes representing the family members’ experience of COVID-19 patients intubated in the ICU are described below.

**Table 2.** Demographic Profile of Patients and Family Members.

Patients (N = 10)	
Age, mean (SD)	54.2 (16.67)
ICU length of stay in days, mean (SD)	24.3 (12.78)
Died during ICU stay or immediately after ICU stay, n (%)	4 (40%)
Family Members (N = 10)	
Age, mean (SD)	50.8 (14.40)
Relationship to patient	
Spouse/partner	3 (30%)
Child of patient	3 (30%)
Parent of patient	2 (20%)
Other relative	2 (20%)
Female, n (%)	8 (80%)
Race, n (%)	
White	4 (40%)
African American	5 (50%)
Asian	1 (10%)
Hispanic, n (%)	1 (10%)
Education	
≤ High school	5 (50%)
≥ College	5 (50%)
Household income	
< 30 K	3 (30%)
30 K-50 K	4 (40%)
50 K-100 K	1 (10%)
> 100 K	1 (10%)
Prefer not to answer	1 (10%)
Employment	
Employed	4 (40%)
Unemployed	3 (30%)
Retired	3 (30%)
Religion	
Christian	6 (60%)
Islam	1 (10%)
None	3 (30%)

### Reactions to the COVID-19 Diagnosis

Family member’s experienced high levels of stress and anxiety after learning about the patient’s diagnosis. They reflected on the past to identify how their loved ones had contracted the virus. Some individuals reported feeling guilty about the events that led up to the diagnosis. One mother expressed regret that she had not discouraged her family member from going ahead with surgery stating: “It may be our fault. When he [patient] went to the hospital to have [an] amputation, he called us and told us about [it].” [Family 20].

Other families reacted by blaming others for the infection, stating that the patient had put themselves at risk, or that they could have done things differently to prevent exposure. One husband thought that his wife (patient) made the wrong decision to travel during the pandemic, which he attributed to be the reason why she contracted the virus.

“This is a pandemic. It is like my wife made [the] wrong decision…she insisted to go there…when she came [back], she came with symptoms.” [Family 21]

Other family members felt that the healthcare system was responsible and perceived that there was a delay in diagnosis and treatment. Their stress was further exacerbated when they felt their loved ones were being dismissed by healthcare providers in the nursing home, and were not being tested for COVID-19 even when exhibiting symptoms.

“They did not do anything about it. They didn’t call the doctor. Then the doctor came and [said he was] clear. Of course, I got a call from the nursing home [later] and said his oxygen level was 60% and had a fever so they sent him to the hospital.” [Family 19]

Family members described how patient symptoms were stressful to witness: “I spoke to her, she barely can talk on the phone…and she said ‘I can’t breathe.’” [Family 7]

### COVID-19 as a Destabilizing Force on the Family Unit

The patient’s diagnosis also created significant physical and psychological uncertainty for the family. While being concerned for the health and wellbeing of their loved ones, family members also felt vulnerable and worried about their own potential for contracting the virus. Two family members had physical symptoms of COVID-19 and tried to advocate for themselves, yet they did not receive attentions from healthcare providers:

“When my mom [got] sick, I [got] sick. I can’t smell anything. My breathing is difficult and I have [a] fever. I called my doctor. The nurse said the doctor will call me but my doctor didn’t call me back.” [Family 1]

Overall, family members were not recommended for testing nor did they receive education on the risk of infection to other family members. One family member stated how her husband struggled from sequela of COVID-19, such as constant shortness of breath, nerve pain, and PTSD. His traumatic survival experience and unpredictable future health worsened their anxiety and suffering:

“He is on tons of medication you know…complications are ongoing…he says he has nightmares. He said he does remember some things that happened to him while he was there, but other things he doesn’t remember. He’s just absolutely terrified.” [Family 17]

Family members also expressed fear and felt powerless due to the unknown outcomes of COVID-19. Emotional distress and negative emotions were evident as family members struggled to adjust their family structure without the patient and relationships between family members were strained. One husband could not sleep at night because he worried about his wife (patient) and their 7 children.

“Kids like always ask me is mom going to come back home? And I don’t have the answer for them. When we do, like video call, some of them have tears in their eyes which made me feel like…it is not good. It made me…very stressed.” [Family 21]

One interviewed family member described being angry because she was estranged from her sister who initially proclaimed healthcare proxy of the COVID-19 patient and she did not share information about the patient’s condition.

“Whatever they told [my sister], I don’t know. I haven’t talked to [my sister] yet. Because me and [my sister] don’t get along. She signed up for that and she said that I want to be mama’s proxy.” [Family 15]

### COVID-19’s Effects on Bereavement Outcomes

In this study, 4 caregivers were bereaved after the patient’s death from COVID-19 created lingering psychological repercussions for the family because they did not have the opportunity to grieve properly or participate in EOL care. One mother experienced complicated grief after her daughter’s death from COVID-19 because she did not have the chance to say goodbye and could not have a “normal funeral.”

“I never got to say goodbye. That is why it is so hard because once you have the funeral…it’s over and you can get, you know, you can sort of deal with that, but when there’s no funeral, no normal…it is like it’s still hanging in the air waiting to happen, you know, I can’t get over [it] the same as you would a normal burial.” [Family 3]

Family members’ complicated grief escalated if the patient did not receive end-of-life care before he/she died from COVID-19 in the hospital.

“She was supposed to going to hospice care and what happened is the doctor called me and told me you know that she [patient] was very ill and was going downhill. He said to me ‘what did you want to do?’ I said, ‘I don’t want her to suffer’ because she really was suffering, and I said ‘I’d rather we just let nature take its course.’ I didn’t want to put her back on the ventilator, then taken off the ventilator so that was it. In the very next day, the day after that, she died so she never got to go to hospice. You see what I mean. We never got to do anything. She was delirious.” [Family 3]

### Desperately Seeking Information

In the absence of being able to observe and participate in patient care because of visitation restrictions, receiving information from others became vital for family members. Family members shared stories of frustration over communication with healthcare providers whom they looked to for information. Although not unique to the COVID-19 setting, it was not the pandemic itself that family members could not understand, but rather that medical language was hard to comprehend.

“The doctor said my mom needs a tracheostomy. And I mean, not right now. Tube is in the mouth. Now [we] can’t do tracheostomy because my mom has pneumonia. When the doctor doesn’t give my mom anesthesia, my mom does not respond. My mom has neurology [consult] and CT scan. The paper, my mom’s little diagram, echocardiogram, I don’t know what does it mean.” [Family 1]

As family members could not be at the bedside to observe the care provided to their loved ones, they did not feel informed despite interactions with healthcare providers. Family members did not feel informed and this made them suspicious about the quality of care for their loved 1.

“They should be honest…not stick around to tell me…we are doing this test, that lab test without tell[ing] me she’s got COVID…that’s [the] number one thing, they have to be very honest and clear about what’s wrong, and what is the plan if they don’t know, and then say we don’t know yet but we are doing that test, we will let you know what we find out. That is it.” [Family 3]

Because they felt uninformed, family members described turning to television and social media to understand their loved one’s care. Television reports about the pandemic became the yardstick for determining good care or understanding what was happening. Family members felt that they had good care when updates were similar to what they were hearing on television. However, reliance on television updates added to their anxiety when what they were hearing from providers conflicted with what they were seeing on the news.

“They have been pretty forthcoming with information. They have been ahead of time. We saw steroid use on the television and they said they already tried. Everything we saw [on] the television and they are top of it.” [Family 20]

“I learned about it from the Facebook. I learned about the people in the hospital and they want to put them on the ventilator. They don’t want to be put on the ventilator but they still survive. They survive because they stay on oxygen. I also heard from the news on the TV.” [Family 14]

Family members were frustrated over the volume of healthcare providers involved and had difficulty keeping information from different team members straight. Participants described the stress of trying to get information and from receiving information from multiple sources that was not always consistent. Such lack of control was the driver of doubt and feelings of uncertainty and mistrust:

“How I am going to make decisions if I’m getting different stories? Where you can feel…you know this person a little bit so they are telling you the truth. I just don’t know who to trust and I just don’t feel like I have any control.” [Family 3]

### Family Member Needs

All of the family members interviewed described ways that they could be more informed about COVID-19 care and treatment. The most common needs were for more timely and regular care updates, and exceptions to allow face-to-face visits despite isolation policies and visitation restrictions. The participants also noted the need for receiving consistent information using different modes such as phone calls, text messages, or a dedicated online patient portal to received daily updates.

“I’d much rather talk to the nurse every day and then have the doctor call me [with] an update and I think that should be done on a regular basis.” [Family 3]

“They [doctors and nurses] are busy…but I just wish that they can keep you updated more often. People are calling them…being aware [we are] waiting…I just feel like they could call you more often.” [Family 2]

Although family members understood the vital importance of isolation, they also believed that there should be exceptions, especially when the patient was dying. They felt untethered from their dying loved ones and believed that at least one family member should be allowed to visit while wearing personal protective equipment.

“I was thinking about what I told the nurse a couple of days before that and she said, well if she gets [to the] the point where she’s dying you could come maybe and put everything on and see her through glass. But I said I don’t know if I’d wanted to see her that way, you know, I can’t even touch her hand or anything even with gloves on. What is the point if I can’t even lean over and tell her mommy is here? How could I possibly do that on the other side of a glass window? I can’t so that was out.” [Family 3]

### Conflicting Feelings About Video Calls

Because family members could not see, touch, or hold their loved ones in the ICU, video calls were introduced to allow family members to virtually visit the patient. However, some family members described that seeing their loved ones virtually contributed to their suffering. One family member described how she cried when she saw the patient lying in the bed, unconscious and connected to the machines. One man who had 7 children, explained that he had to stop having video calls with his wife who was intubated because it was so disturbing to his children.

“It was not necessarily a positive or pleasant experience. People were saying you feel so much better because you will be able to see him. I did not find that and I did not get any comfort from it.” [Family 20]

Family members were conflicted about video calls because they wanted the opportunity to see the patient, yet the images of their loved ones lying there was upsetting. However, family members who were not able to have video calls shared their deep disappointment.

“I wish that we would able to either do video conference but their computer system and my computer system are not compatible. Most importantly, I wish we could’ve been there with him going through that living with them being there while he handles procedures [being] done.” [Family 19]

“Right now, video talk is not helping. If the person sleeps, the nurse [is] just showing you the face. That makes you stressed. We haven’t heard voice and we haven’t talked. She is not opening her eyes. If you compare to when she was healthy, it will take you back. It makes you feel things [are] not normal.” [Family 21]

### Appreciation of Care

Despite the uncertainty, all family members expressed appreciation for the providers and recognized their sacrifices. They worried about burdening them too much and felt respected and supported during interactions:

“I mean I really appreciate any information passed on to us…since they call me really aware that [there] is a family waiting.” [Family 12]

“Because they are busy. Many patients, you know it is a lot. Right now, [it] is really hectic there. I just wish that I could know more about my aunt. I understand that they got other people to take care and…[it is] stressful on them too.” [Family 2]

Family members had pleasant experiences when they talked to healthcare providers because they felt respected, supported, and felt they could control the patients’ care.

## Discussion

Given the contagious nature of COVID-19, the Center for Disease Control and Prevention (CDC) recommends limiting visitors to ICUs,^7^ resulting in a paradigm shift in ICU care. In this study, the experiences described by family members about their loved ones being diagnosed with COVID-19 and ventilated in the ICU included: (a) reactions to the diagnosis, (b) a destabilizing force on the family unit, (c) incomplete bereavement, (d) a desperate search for information, (e) communication needs with healthcare providers, (f) conflicting feelings about video calls, and (g) appreciation of care. Family members of COVID-19 patients in the ICU needed support (“family-centered care”) and assessment of their emotional state, which could be provided through telephone calls and home visits with the palliative care or social work team providing active listening, reassurance, empathy, and networking.^11^

Although evidence shows that family members want proactive and structured communication,^12-17^ this study found communication between healthcare providers and family members were insufficient. Family members reported difficulty reaching providers and reported feeling uninformed, which suggests that various approaches for communicating with families should be considered. Moreover, family members had low health literacy about ICU care which has been shown to contribute to feelings of mistrust and doubt in the quality of care.^1^ In a pandemic, a component of patient/family education about the disease must also address misinformation and myths procured through social media and television.

Visitor restrictions in ICUs adversely affected patients and their family members. In order to counteract this issue, video calls were introduced to facilitate communication between patients and family members when they were restricted from seeing their loved ones in the hospital.^18-20^ However, in this study we found that many family members were shocked and felt more stressed after they saw their loved ones intubated, sedated, and connected to the ventilator.

### Clinical Implications

The patient mortality rate in this study was 40% which was similar to national rates.^21^ Clearly, there was a significant need for high quality EOL care and effective communication with family members of COVID-19 patients in the ICU.^22-24^ These needs may have been met more effectively with an early referral to palliative care. The role of palliative care for COVID-19 patients in the ICU has been well established.^25-27^ Palliative care approaches provide opportunities to initiate goals-of-care conversations with family members to help them cope with uncertainty.^27^ Moreover, the multi-disciplinary approach of palliative care facilitates the support of family members (i.e., providing frequent medical updates, social support, and enhancing communication with healthcare providers),^25^ and the utilization of medications to manage pain and other distressing symptoms (i.e., anxiety and delirium)^26^ may alleviate both patient and family suffering.

In this study, family members of ventilated COVID-19 patients were not able to be at the bedside to advocate for their family member. Because families had a harder time understanding the gravity of the situation, and were not confident whether their loved ones were comfortable and being well cared for, they may have been more likely to want to do everything to fight for survival. Prior research suggests that families of patients with COVID-19 are less inclined to discuss goals of care and withdrawal life support than non-COVID-19 patients with terminal illness.^22^ Communication strategies to support isolated family members based on the findings of this study are summarized in Table 3.

**Table 3.** Recommendations for Communication Strategies to Support Isolated Family Members.

1	Conduct an initial assessment of communication preferences with family members, such as determining who is the proxy or surrogate decision maker for the patient
2	Ask for family preference or no preference for seeing patient ventilated through video call and prepare family for what they will see when the patient is on ventilation
3	Keep family members informed at least once a day and at an agreed time^18^
4	Use plain language and give correct information to help family members understand the situation and gain feelings of control
5	Hold ongoing discussions with family members about emerging clinical scenarios to foster shared decision-making
6	Expect that family members will often ask the same questions continually because they need to receive information repeatedly and in different ways^24^
7	Reassure family that their loved 1 is being treated as a whole person rather than solely as a critically ill patient^28^
8	Hold family meetings via telephone or videoconference as early as possible, ideally within 5-7 days of ICU admissions^28^
9	Initiate palliative care consultation upon admission to the ICU, assess family readiness and emotional state^11,23^

### Strengths/Limitations

This is the first study providing close insights into the experiences of family members who could not visit their loved ones in the ICU. A key strength is the rigor used to capture the experiences of family members of ICU COVID-19 patients. However, several limitations should be noted. As an exploratory qualitative study, the sample size was small (n = 10), and family members were only selected from a single hospital in the northeastern United States. Despite sharing the common ground of feeling anxiety, stress, and uncertainty among family members, there will likely be experience variation across racial and geographic populations, and caution is required with interpretation. Likewise, although one Burmese family member was interviewed, we have not been able to capture a broad representation from different cultural or non-English speaking backgrounds. These groups may have different experiences and support needs of family members of ICU COVID-19 patients which warrants future study.

It must also be acknowledged that only one bereaved family member participated in the second interview (4-6 weeks after the ICU discharge). This limits our ability to comment on the chronicity of family members’ experiences when their loved ones died from COVID-19 in the hospital. In addition, despite recruiting first degree relatives who were listed in the medical record as the person of contact for the patients, given the “no visitor” policy, only surrogate decision makers’ contact information were documented in the medical record. Hence, we only interviewed surrogates due to pandemic conditions. This did not represent other family members, and future research should address larger family systems.

## Conclusion

The findings in this study contribute a valuable in-depth understanding of urgent needs for family members of ventilated COVID-19 patients in the ICU. It has been predicted, and indeed, we have seen other highly contagious diseases surface, that some effort should be put into mitigating the changes presented by these findings. Future work will need to consider the implementation of palliative care and communication strategies aimed at recognizing individual family member’s burdens and preferences. The current study highlights the substantial stress, anxiety, and uncertainty of family members of COVID-19 patients and early palliative care referral should become a priority to improve communication and support grieving for family members. Our findings can be used to guide palliative care and other providers caring for ICU COVID-19 patients and their family members.
